# Biomanufacturing of Natural Bioactive Compounds

**DOI:** 10.3390/molecules31071102

**Published:** 2026-03-27

**Authors:** Honglei Liu

**Affiliations:** School of Basic Medical Sciences, Shandong University, Jinan 250012, China; lhl@sdu.edu.cn

Natural bioactive compounds (NBC) have been research targets for decades due to their various usages in agriculture, industry, and more importantly, pharmaceuticals [1,2]. Over 200,000 secondary metabolites produced by nature’s chemists—mainly plants and microbes—contribute significantly from academic curiosity to sustainable development and human health protection [3]. The development of modern pharmaceuticals relies greatly on the production of bioactive compounds, with approximately 60% of antitumor and anti-infective drugs being extracted or derived from natural metabolites [4]. However, the production of many active compounds in natural plants is usually scarce, involving complex metabolic processes. Thus, leveraging modern chemical or molecular biological techniques to enhance the production of precursors or target molecules in NBC biosynthetic pathways remains a core priority for obtaining industrial and pharmaceutical materials [5,6]. The contributions featured in the Special Issue “Biomanufacturing of Natural Bioactive Compounds” indicate the recent advances in multiple research aspects, including metabolite profile analysis aiming to elucidate the biosynthesis pathways, heterologous biosynthesis of NBCs, and impacts of other compounds on NBC stability; the plant or microbial chassis used in the biosynthesis of NBCs; as well as the comprehensive analysis of the production, applications, working mechanism, and therapeutic effects of specific NBCs.

The foundational step for NBC exploration is to discover their natural existence and clarify their biosynthetic pathways in the natural organisms. Three contributions in the Special Issue focus on the metabolite profiles of *Lonicera caerulea* (blue honeysuckle), *Sphingopyxis* sp. and *Piper aduncum* [Contributions 1–3]. Razgonova et al. examined honeysuckle samples collected from four regions of Russia that covered almost the entire Eurasian continent and identified rich polyphenolic bioactive compounds via LC-MS and MS/MS approaches, which provide extensive bioactive additives for pharmaceutical and nutritional research, facilitating the development of functional products. Liu et al. investigated the biosynthesis of carotenoids in *Sphingopyxis* sp. USTB-05 using metabolomic analysis, identifying seven carotenes and six xanthophylls. Notably, Zeaxanthin was in particular rich, with a content of 37.1 µg/g dry cells, which greatly improved our knowledge of *Sphingopyxis* sp. in carotenoid biosynthesis. de Luna et al. characterized the chemical diversity of the bioactive fractions of cultivated *Piper aduncum* extracts and investigated their anti-*Mycobacterium tuberculosis* activities. Through LC-MS/MS analysis of the most active partition from the ethanolic leaf extract, 83 compounds were identified, and the antimycobacterial chemical fingerprint was built.

Traditional extraction methods are far from meeting global societal demand. Extraction of NBCs from plants requires intensive resources, and chemical synthesis of the compounds often leads to complex separation processes and heavy pollution of the environment. Additionally, large-scale collection has also left some medicinal plants on the brink of resource depletion [7,8]. Synthetic biology is rewriting this paradigm with many benefits: safety in medical supply, sustainability in agriculture and forestry, and environmental friendliness [9]. Many successful cases have demonstrated the achievements worldwide. For example, researchers from the Kunming Institute of Botany identified one of nature’s largest biosynthetic gene clusters (BGCs) in *Astragalus membranaceus*, enabling the production of astragaloside in tobacco at a yield of 2.224 mg per gram of dry weight, which eliminates the reliance on 4–5-year-old *Astragalus* crops and reduces the use of arable land [10]. In 2024, Astolfi et al. achieved a landmark advance in the biosynthesis of QS-21, the only approved human anticancer and anti-infective vaccine adjuvant, raising the production by engineered yeast to 100 μg/L via a 3-day fermentation using glucose as the sole carbon source, while the natural content is only 0.0032% (*w*/*w*) dry weight in the bark of *Quillaja Saponaria*. The research introduced 38 exogenous genes from six biological sources and reconstructed one of the longest heterologous biosynthetic pathways with similar plant subcellular compartmentalization in *Saccharomyces cerevisiae* [11].

Every advancement in the field of biosynthesis, from the identification and improvement of NBC-producing organisms, the expression and modulation of exogenous genes, and the synergistic optimization of gene networks in cell factories, to the efficient, green, and large-scale production of bioactive compounds, promotes the upgrading of both the bioactive substance and pharmaceutical industries. Within this Special Issue, Si et al. [Contribution 4] summarized the diverse natural antimicrobial active substances identified in *Pseudomonas aeruginosa*, highlighting the bacteria as an outstanding resource to find novel pharmacologically important NBCs, which may have great potential to identify novel drugs against infections caused by drug-resistant pathogens. Huang et al. [Contribution 5] generated a *Pseudozyma* sp. P4–22 mutant through atmospheric and room-temperature plasma mutagenesis, which efficiently produces squalene in cost-effective culture media, achieving a biomass of 64.42 g/L and a squalene yield of 2.06 g/L, respectively. This study provided a promising strain for the production of squalene on a commercially efficient scale. Yin et al. [Contribution 6] reviewed the biosynthesis of phosphoenol pyruvate–oxaloacetate–pyruvate-derived amino acids (POP-AAs) and suggested that, to obtain higher yield and productivity with maximum theoretical values, reconstruction of novel heterologous metabolic and regulation pathways to improve carbon flux redistribution in the POP node might be the key factor. Han and Miao [Contribution 7] compared the heterologous biosynthesis of plant secondary metabolites using plants or microorganisms as chassis, noting that some complex bioactive compounds are still unable to be produced successfully in microorganisms due to unclear metabolic pathways, low enzyme activity, and interference with endogenous metabolism. Compared to plant chassis, microorganisms have garnered increasing attention due to their faster growth rates, smaller and more well-characterized metabolic networks that are amenable to genetic manipulation, and simpler cultivation conditions. Hashim et al. [Contribution 8] contributed a review exploring the biomanufacturing of some bioactive compounds for periodontal disease management, describing production processes involving microbial fermentation, plant cell cultures, and enzymatic synthesis.

Stable production is a crucial prerequisite for industrial biosynthesis. Many metabolites and intracellular intermediates can influence the stability of the final products. Gao et al. [Contribution 9] compared the effects of different thio-containing compounds, which are abundant in radish seeds, on the stability of sulforaphene (4-methylsufinyl-3-butenyl isothiocyanate, SFE), a promising cancer-preventive compound. Their results showed that sulfide, glutathione, and cysteine all modulate the degradation rate of SFE, with sulfide and glutathione exerting the most significant effects. Furthermore, combined treatment with an adequate concentration of sulfide and seed heat treatment effectively enhanced SFE production. These findings provide valuable insights for improving both the yield and stability of SFE for industrial applications.

The diverse pharmacological functions of NBCs make them an important arsenal for humans to fight against diseases. Countless revolutionary drugs have been developed using bioactive compounds as lead prototypes. However, for a long time, NBC research was largely driven by accidental discoveries: the biological functions of many compounds were identified, yet their underlying molecular mechanisms remained elusive [2]. The complexity and diversity of NBCs pose great challenges during elucidating their potential targets, which are becoming a bottleneck hindering the clinical translation of these active compounds [12]. In this Special Issue, Hashim et al. [Contribution 10] overviewed the molecular mechanisms of some bioactive compounds in the treatment of periodontal disease, summarizing several key regulatory pathways involved in their therapeutic effects. This may provide guidance for comprehensively understanding the action mechanisms of NBC and for selecting promising drug target molecules.

The 10 contributions in this Special Issue collectively reflect the important recent progress in NBC biosynthesis and biomanufacturing research, which holds considerable significance for pharmaceutical innovation. With the rapid development of cutting-edge technologies, the research of natural product-based drugs is undergoing comprehensive innovations in some areas, such as AI-associated intelligent drug discovery, target identification, and mechanism clarification as well as large-scale production by synthetic biology. The discovery of new drugs will take on increasing significance from the exploration of new NBCs.
